# Gendering strategic action fields in sports governance

**DOI:** 10.1177/10126902221136084

**Published:** 2022-11-14

**Authors:** Madeleine Pape, Lucie Schoch

**Affiliations:** 27213 University of Lausanne, Switzerland

**Keywords:** gender, organization, field, masculinity, leadership, barrier

## Abstract

How do meso-level field relations shape the ways that sports organizations act on gender equality? In this paper, we approach international sports governance as comprised of meso-level fields of strategic action in which male dominance and relations of masculinity are centrally at stake. We focus on the *Union Cycliste Internationale* (UCI), showing how the organization's efforts to address gender inequality are shaped by its relations with adjacent actors in the field. These actors jockey to form strategic coalitions as they struggle over the influence and resources to define the field configuration of international cycling, with challenges to the gendered status quo requiring careful management. Based on semi-structured interviews with individuals who held an elected or staff position within the UCI between 2005 and 2020, we show how field relations shaped the work of the UCI Women's Committee during this period as well as the experiences of women who succeeded in accessing decision-making roles. The UCI emerges in our analysis as a central governance unit via which the historical accumulation of advantage to men is preserved. We suggest that studying meso-level fields of strategic action can advance sociological research more broadly on how sports organizations are shaped by their contingent, dynamic, and (gender) unequal context.

Sociologists of sport have excelled in identifying the organizational dynamics that contribute to persistent gender inequality in the practice and governance of sport (Adriaanse and Schofield, 2013; Claringbould and Knoppers, 2012; Elling et al., 2018; Piggott and Pike, 2019). From the effects of formal policies, to the informal and unwritten elements of organizational culture and male-dominated political networks, to the role of gender stereotyping in the selection of decision-makers, the organization is a key unit in the reproduction of gender relations in sport (Henry and Robinson, 2010; Hoeber, 2007; Hoffman, 2011; Hovden, 2010; Schull et al., 2013; Shaw and Hoeber, 2003). Much of this scholarship recognizes that sports organizations do not operate in a vacuum: rather, they are located within sport as a wider macro institutional setting, which is characterized by a gendered logic that constructs sport as a symbolic and material stronghold of men and masculinity (Burton 2015; Evans and Pfister, 2021; Piggott and Pike, 2019; Schull et al., 2013). However, sociologists of sport have less often focused on the specific means by which sports organizations are shaped by their more immediate governance context, and with what consequences for the pursuit of gender equality initiatives and the experiences of women who reach leadership positions.

This paper offers an innovative approach that examines meso-level field dynamics, showing how they impress upon and shape gender relations at the organizational level, with consequences for gender equality in sports governance more broadly. Looking to the case of international cycling, we build on integrative approaches to gender theory that conceptualize gender relations as embedded in and reproduced at the micro, meso, and macro levels of social life (Acker, 1990; Connell, 1987; Risman, 2004). We focus specifically on enlarging the meso-level analytical agenda for studies of gendered sports organizations, elaborating in particular the under-studied role of inter-organizational context—that is, the dynamic and interdependent relations between adjacent organizations with a stake in a given issue––in shaping practice and policy. To do so, we draw on a meso-level theoretical approach that has rarely been used within sociology of sport despite its influence in sociology more broadly: namely, the strategic action fields (SAFs) theory of Fligstein and McAdam (2011). We ask: how do meso-level field relations shape the ways that sports organizations, and specifically International Federations (IFs), act on gender equality? Additionally, what do these field relations mean for the experiences of those women who succeed in accessing decision-making roles?

At the center of our analysis is the *Union Cycliste Internationale* (UCI): the IF and peak governing body for the sport of cycling. The UCI is significant as one of the oldest IFs in the Olympic Movement, which in recent years has sought to demonstrate a commitment to redressing a long history of male dominance both on and off the playing field. This has included sequential UCI Presidents being elected on platforms that have centered the advancement of women's cycling. Concrete actions taken by the UCI have included the establishment of a Women's Committee as well as an external assessment of how the UCI as a workplace has addressed gender equality amongst its own staff. Given that the composition of the UCI elected leadership––and the actions it takes––are shaped by adjacent meso-level actors such as Continental Federations (CFs) and National Federations (NFs) (Chappelet et al., 2019), the UCI offers a useful case for exploring how SAF theory can aid in conceptualizing the field-like nature of sports governance.

Building on Fligstein and McAdam (2011), we show how different actors are unequally located within the field of international cycling and how they jostle with one another as they defend the status quo or seek to reconfigure field relations. We show further how, though absent from Fligstein and McAdam's original conceptualization and its wider application, gender is a key organizing logic that explains the configuration of SAFs. We contend that male dominance and relations of masculinity continue to be centrally at stake in the SAF of international cycling, with consequences for both how gender equality initiatives are implemented by the UCI and what women experience when they succeed in reaching decision-making positions.

In what follows, we summarize how meso-level approaches to gender relations have been used to date to explain the gender dynamics of sports organizations, particularly Raewyn Connell's (1987) notion of gender regimes and Joan Acker's (1990) concept of gendered organizational logics. Turning to Fligstein and McAdam's (2011) SAF theory, we then develop a meso-level approach to sports organizations to aid in more precisely conceptualizing how a single organization is shaped by its gendered inter-organizational field context. Next, we empirically demonstrate how international cycling governance operates as a gendered SAF in which relations of masculinity are centrally at stake, before focusing on the implications of this for the UCI Women's Committee and for women in UCI leadership roles. We conclude with the implications of our approach for sociological studies of the meso-level mechanisms that continue to undermine the advance of gender equality in sport.

## The meso-level politics of gender

According to the “integrative tradition” within sociology of gender (Risman, 2004: 430), gender is more than the property of an individual person or an interactional achievement: it is an ideology and organizing logic that is built into and reproduced via broad macro institutions like the state, education, family, the economy, and sport (Connell, 1987; Lorber, 1994; Martin, 2004). It is also reproduced at the meso-level, via organizations whose structures and practices are characterized by complex ideologies and relations of gender––what Acker (1990) has referred to as a gendered “organizational logic” (Acker, 1990: 147). Acker's work has profoundly influenced feminist scholarship on the organizational dynamics of sports leadership and governance, which has shown how the logic of sports governing bodies frequently rests upon an ideology of fundamental (and unequal) gender difference (Adriaanse and Schofield, 2013; Hoeber 2007; Pape, 2020; Schull et al., 2013). Symbolically, sports organizations continue to associate leadership with men and masculinity while devaluing attributes deemed to be feminine, with the result that women must over-perform relative to their male counterparts to be considered for leadership roles (Burton et al., 2011; Claringbould and Knoppers, 2007; Hovden, 2010; Piggott and Pike, 2019; Shaw and Hoeber, 2003). As sports organizations move away from the overt exclusion of women and towards a stated commitment to gender equality, informal practices can continue to undermine the advancement of women, such as subtle discursive regimes that valorize masculinity, or vague definitions of gender equality goals that eschew meaningful action and accountability (Claringbould and Knoppers, 2012; Hoeber, 2007). However, though it has proven useful to sports scholars, Acker's approach to theorizing gendered organizations does not extend beyond the boundaries of a single organizational unit.

A second meso-level approach that has proven useful for sociologists of gender and sports governance has been that of Raewyn Connell, whose concept of “gender regime” (1987: 120) describes “[t]he state of play in gender relations” (p. 199) in a given organizational or institutional setting that, like Acker, encompasses the interactional, symbolic, and structural reproduction of gender. However, unlike Acker, Connell's approach conceptualizes the meso-level as encompassing not only single organizations but also their immediate institutional context. Connell contrasts this idea of institutional context at the meso-level with her macro-level notion of the “gender order”: the much broader “complete structural inventory” of multiple gender regimes and the relationships between them, which together form a “macro-politics of gender” on a “society-wide scale” (p. 139). However, what the inter-organizational institutional context is, and how it matters to gender dynamics at the meso-level, is not well defined in Connell's theory.

Existing feminist analyses suggest that factors external to the sports organization are significant to understanding its existing gender regime. For example, numerous studies point to the role of gendered networks in perpetuating male dominance of leadership roles (Hoffman, 2011; Piggott and Pike, 2019; Schull et al., 2013; Shaw, 2006). While this can refer to relations between men within a given organizational setting (Claringbould and Knoppers, 2007), networks beyond organizational boundaries also appear to be significant. Schull and colleagues’ work (2013) is notable in showing how the appointment of an athletic director in collegiate sport in the United States is shaped by the male-dominated political and media networks that extend beyond the university itself. In this case, the “brotherhood” (p. 66)––an inner circle of male stakeholders aligned with men's collegiate sport––used their connections with the local media to ensure a woman was not appointed to the position of athletic director. Building on this work, we identify a need to enlarge the meso-level research agenda in order to better understand how the gender dynamics of individual sports organizations are shaped by their immediate inter-organizational context, including at the international level, where sports organizations are frequently interdependent (Geeraert et al., 2015).

With this theoretical agenda in mind, we suggest that understanding the actions of the UCI on gender equality, and the experiences of women in UCI leadership roles, requires attention to the full range of meso-level mechanisms that shape the governance of international cycling. We expect further that gender relations are at stake in and condition relations between actors in this meso-level field as they jostle for influence and resources. Relatedly, and given the longstanding coupling of cycling with men and masculinity (McLachlan, 2016), the preservation of (white, heterosexual) male privilege vis-à-vis women will likely be central, as will the contested and uneven distribution of power among men (Anderson, 2009). As Connell observes, “What reasons for change have enough weight, against this entrenched interest, to detach heterosexual men from the defense of patriarchy?” (1987: xiii). To aid in conceptualizing these gendered meso-level inter-organizational dynamics, we now turn to scholarship on the networked relations of international sports governance and develop an account of international cycling governance as a field of strategic action.

## Strategic action fields as a meso-level framework

Governance scholars within sports studies have debated how to conceptualize the structure of sports governance and the role of international sports governing bodies therein (Geeraert and Bruyninckx, 2014; Geeraert et al., 2015; Meier and Garcia, 2021). Such work has challenged the traditional view of sport as an autonomous domain of governance, within which international governing bodies are autonomous entities that wield considerable top-down influence within a vertical chain of command (Croci and Forster, 2004; Geeraert and Bruyninckx, 2014). Influenced by theories of networked governance, contemporary scholars instead understand sports governance as “a web of relations between international sports governing bodies, state, business, and civil society actors,” in which international governing entities like IFs “lose their ability to hierarchically govern their respective sports” (Geeraert et al., 2015: 483). Whereas such work has often focused on how entities *external to sport* impress upon the policymaking efforts of sports governing bodies (Croci and Forster, 2004; Geeraert et al., 2015; Meier and Garcia 2021), we attend to how the interdependent relations amongst adjacent actors *within* the institution of sport are significant to how a single organization, such as an IF, can govern (Chappelet, 2016).

SAF theory is ideally suited to conceptualizing these meso-level inter-organizational dynamics. According to Fligstein and McAdam (2011), SAFs serve as “the fundamental units of collective action in society” (2011: 3): the structured fields of action within which social actors interact with one another as they compete for power and influence, form strategic alliances and collective identities, and seek to redefine or defend the organization of the field. Like Connell's (1987) account of gender regimes, in which gender politics are characterized by conflict and the possibility of change, Fligstein and McAdam emphasize that fields are dynamic, situational, and contextual, with their boundaries and influence on each other shifting depending upon “the definition of the situation and the issues at stake” (p. 4). Some networked approaches to sports governance similarly recognize the potential for conflict and change, such as Meier and Garcia (2021), who note that network structures are not necessarily stable, collaborative, or equitable. SAF theory centers these contingent, unequal, and conflictual relations from the outset, understanding contestation and alliance formation as key to how trajectories of policymaking and (resistance to) social change unfold.

Three key elements of SAFs are particularly useful for our analysis. First, SAFs are comprised of diverse sets of actors that have varying degrees of power over the field structure and associated interpretive frames. Fligstein and McAdam (2011) characterize these actors as the *incumbents*, who wield disproportionate influence and whose interests tend to be heavily reflected in the organization of the SAF; the *challengers*, who occupy less privileged positions and, given different interests and a different views of the field compared to incumbents, may look to change the current order; and the *governance units*, those actors charged with overseeing compliance with field rules, who tend to reinforce the dominant logic and safeguard the interests of the incumbents (Fligstein and McAdam, 2011: 5). Governance units can typically be considered “defenders of the status quo” and a “conservative force” during moments of conflict within the field (Fligstein and McAdam, 2011: 6).

A second element concerns field boundaries. According to Fligstein and McAdam, fields are not autonomous, self-contained worlds: rather, they are located within a complex web of hierarchically nested fields. Some adjacent SAFs may be more proximate than others and regularly exert an influence on the field in question. For example, the Olympic Movement overlaps the sport of cycling and is able to influence cycling actors, particularly via the International Olympic Committee (IOC). This resembles what Jean–Loup [Chappelet (2016)] has described as the overlapping domains of the “regulated Olympic system” (the bodies that directly govern Olympic sports) and the “total Olympic system” (including sponsors, the media, and local sports clubs). What SAF theory adds is a dynamic understanding of field formation and their overlapping boundaries, including within a single sport. Which actors are relevant and how they are positioned relative to one another will depend upon the issue at stake. The SAF that forms around doping in the sport of cycling, for example, would implicate different sets of actors and power relations than the issue of grassroots cycling development in a particular country. In each case, the UCI would be relevant to the extent that the issue is successfully framed as warranting the organization's attention and resources.

This leads to the concept of “social skill,” understood as the ability to enroll other actors in one's agenda for the field, which is key to Fligstein and McAdam's understanding of collective and strategic action (2014: 6). Strategic action is defined here as the “attempt by social actors to create and maintain stable social worlds by securing the cooperation of others” (p. 7). According to Fligstein and McAdam, successfully doing so requires an actor to have a highly developed capacity to “read” their field environment, frame their desired line of action accordingly, recruit other actors in service of this frame, and engage them in collective action. This way of conceptualizing collective action distinguishes Fligstein and McAdam's approach from other field theorists, including Bourdieu (see Fligstein and Vandebroeck, 2014). Collective and individual actors with roughly the same power can agree to a political coalition, often by creating a collective identity and usually under a compromise. This differs from Bourdieu's (1977) theory, which is relatively silent on the question of how collective action and cooperation are achieved in the context of a field structure characterized by power and hierarchy. SAF theory further departs from Bourdieu in emphasizing the heterogeneity and contingency of field formation, with strategic field dynamics depending on the issue at hand and how the interests and relationships between relevant actors are articulated.

However, neither networked accounts of sports governance nor SAF theory account for the role of gender. According to Connell, the configuration of a gender regime will reflect the dynamic “articulation of interests and organization of political forces” around hegemonic definitions and relations of gender (1987: 139). We argue that gender will serve as an organizing logic that shapes the structure of SAFs and the frames that are possible to articulate within them. Field relations within international cycling––what's at stake, who wields disproportionate influence, and whose interests are marginal to the organization of the field––are unlikely to be gender neutral. Configurations of actors and their “shared understanding” of field rules––“what tactics are possible, legitimate, and interpretable” (Fligstein and McAdam, 2011: 4)––will likely reflect (and reproduce) male dominance. “Social skill” will likely be imbued with ideologies and relations of masculinity, which will shape access to the discursive and material resources needed to enroll actors behind a given agenda. Masculine incumbents may at times be so absorbed by struggles over power amongst themselves that even capturing their interest will be a challenge for those in more marginal positions. In what follows, we examine the UCI as one example of how these gendered field relations could operate in practice.

### The *Union Cycliste Internationale* as case study: Data and methods

IFs are diverse organizations that vary widely in their progress on gender equality in leadership and on the playing field (Henry and Robinson, 2010; Schoch and Clausen, 2019).^1^ Our case study considers a long-standing Olympic sport that is largely structured by masculine relations. Founded in 1900, the UCI governs eight disciplines of cycling, four of which are represented in the Olympic Games program. Most of these four disciplines are characterized by numerical gender parity on the playing field, with road cycling predicted to reach parity by the 2024 Olympic Games. Unlike Olympic competition, professional road cycling––the sport's most commercially lucrative discipline––is not subject to the IOC's target of gender parity and remains highly inequitable, with women road cyclists long fighting to gain equitable professional opportunities (McLachlan, 2016).

While for many years the UCI made limited progress on gender equality, with a woman only appointed to the UCI Management Committee (executive board) in 2000, it has recently taken stronger action. A Women's Committee (WC) was created in 2013 along with a women's cycling coordinator position in 2014. Equal prize money for women and men at the UCI World Championships was established in 2016. Since 2019, the UCI has required at least 25% “of each gender” on the executive boards of CFs and amongst voting delegates sent to the UCI Congress (UCI, 2019). A woman became Director General for the first time in 2018 and, at the time of writing, 33% of UCI Management Committee members are women, including a second consecutive female Vice President. In 2019, the UCI became the first IF to obtain EDGE (Economic Dividends for Gender Equality) certification, a standard that assesses gender equality in the workplace. Following these developments, and after the completion of our study, in December 2021 the UCI disbanded its WC and restructured the women's cycling coordinator role. However, as we will show, claims of progress on gender equality belie the limited changes within the gendered SAF of international cycling, which continues to be defined by relations of masculinity.

We conducted 22 semi-structured interviews with current or former UCI employees (*n* = 15) and individuals who had held a position within the UCI governance structure (*n* = 7) between the years 2005 and 2020. These were purposively sampled individuals and included one UCI President and four UCI Director Generals. Twelve interviewees were women. Interviewees were asked a range of questions, including regarding their own trajectories, the challenges facing women in leadership roles, how and to what extent the UCI can act on gender equality, and the role of the WC. These interviews were recorded, transcribed, and iteratively coded by both authors according to the principles of abductive analysis (Tavory and Timmermans, 2014). In practice, this meant bringing existing theoretical frameworks to bear on the data and identifying where the interview material pointed to opportunities for new theory building. Our analysis was supplemented by material from UCI documents and media reports, which aided in triangulating the insights from interviews.

Part I of our analysis shows how international cycling governance operates as a meso-level SAF that is characterized by dynamic, contingent, and distributed power relations across actors. We show how these field relations are gendered from the outset, underpinned by relations of masculinity that involve not only the reproduction of male dominance but also power struggles amongst men. In part II of the analysis, we examine more closely how gender figures within moments of instability and conflict within the field. Here, we focus on the Women's Commission (WC) (2013–2021) as a concrete UCI action on gender equality. Building on scholarship showing the contested and limited role of women's committees in international sport (Krech et al., 2022; Matthews, 2021), we show how the UCI WC can be understood as a managed intervention that was constrained by existing field struggles. We also present interviewee accounts of women who have succeeded in accessing UCI leadership roles and, as challengers, have sought to disrupt existing field relations. Like the WC, we show that these women are included to the extent that they accept the existing field configuration––and men's place within it.

## Part 1: Men, power, and cycling governance as a strategic action field

### The networked structure of international cycling

**Figure 1.**
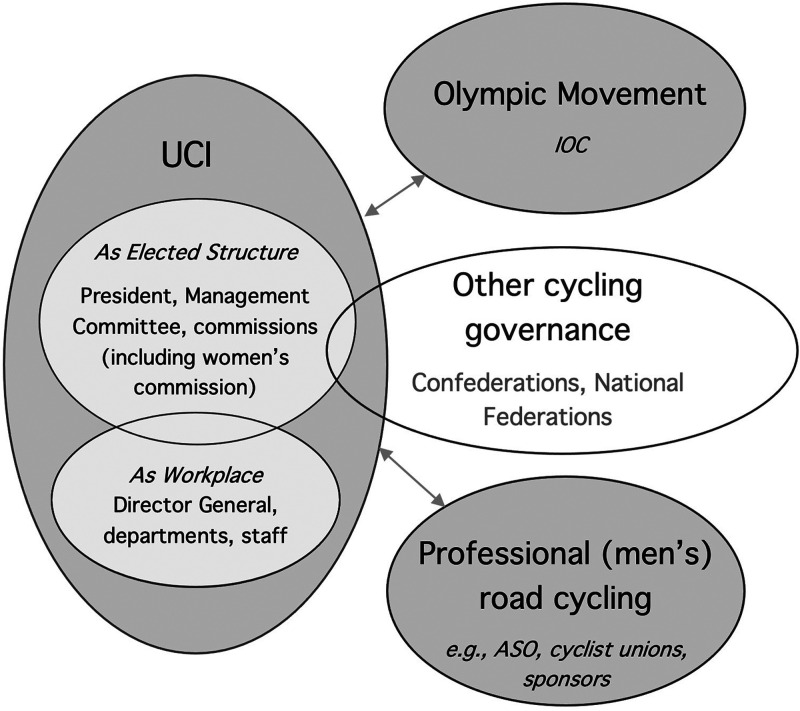
International cycling actors and adjacent SAFs.

When asked to explain gender dynamics at the UCI, interviewees consistently volunteered that the UCI does not operate in a vacuum: that “politics,” the contested, interdependent relations between the UCI and other actors, were always impinging on the autonomy of the UCI and shaping the strategic actions of its decision-makers, including in relation to gender equality (Figure 1). Numerous influential actors were identified, most immediately NFs and CFs, who exert a relatively direct influence on the UCI by deciding the election of the President, shaping the composition of the Management Committee, and nominating representatives for other committees. Indirectly, private race organizers from the field of professional (men's) cycling––for example, the Amaury Sport Organisation (ASO), which organizes the *Tour de France* as well as other renowned races––wields power in the form of rivalry, given the commercial value of their events. Adjacent and overlapping the field of international cycling is the Olympic Movement, led by the IOC, which wields influence over the actions of the UCI given the symbolic and financial significance of Olympic Games participation.^2^ Together, these actors form a “meso-level social order” within which “actors with varying resource endowments via for advantage,” each with a different stake in shaping how the UCI governs international cycling (Fligstein and McAdam, 2011: 3).

### The power of UCI as the central governance unit

Various interviewees debated where decision-making power was located within this SAF According to one former director:

One has to ask the question, who really holds the power [in the sport of cycling]? It's not the Management Committee. The members of the Management Committee are not really the ones who have the power. They see each other three or four times a year and they represent interests that are often divergent from the UCI … The power at the UCI, it's in the hands of the President.

Relative to other IFs, the UCI is characterized as having a strong presidency (Chappelet et al., 2019). Yet the President was also revealed as having limited power, particularly given their electoral reliance on the support of other actors within the field, whose interests must be strategically managed. This male-dominated network is then reflected in the composition of the UCI. According to one former staff person:

[The UCI] is a political organization. It's an organization that depends on votes for the elected president, for the members of the UCI executive committee, who are all presidents of National Federations––[who] are elected themselves. So, there is a complex system behind [cycling governance] that still hasn’t been opened, I don’t think, much to women.

Another interviewee, a former elite cyclist and UCI committee member, similarly highlighted the field-like governance structure feeding into the UCI as gendered: “I think it's the federation model” she replied, when asked what factors impede the advancement of women leaders. A former UCI Director General detailed this process: “In order to change [the under-representation of women leaders at the UCI], you would have to incentivize National Federations to return more women elected officials. But, like I say, there are some things you control, certain things you don't.” Another former Director General explained the UCI could influence the gender equality actions of other actors in the field only “by explanation, by positive motivation, by co-construction.” Within this meso-level social order, then, the extent to which the UCI will act on the leadership decisions taken at the national and continental level is constrained by the interests invested in the (gendered) status quo.

### Masculinity and field stability

The strength of historically contingent relations of masculinity within this governing structure was evident in the number of interviewees that referred to cycling leadership as a “club” and as “old school.” One interviewee described this club as characterized by a “matrix of favors.” A former staff person explained the limits to UCI action on gender equality, stating: it “always goes back to who holds the power, who is going to give votes, who is going to approve budgets, and that ends up being an old boy's club.” Highlighting the relationship between social skill and relations of masculinity, he added: “Ultimately this networking component ends up being more influential than competence.” A former staff person similarly explained that promoting women leaders was “complicated,” “not for reasons of skills, knowledge,” but because “it's just a cultural environment” dominated by “old school men.” One former committee member explained that she didn’t pursue other leadership roles at the UCI because “it's a very conventional, male-dominated sport and organization,” adding “it's a lot of, who do you know? Who can help you?” Consistent with research on masculine networks of sports leadership (Hoffman, 2011; Schull et al., 2013; Shaw, 2006), men in leadership positions benefit from their position as incumbents, which enables them to build meso-level coalitions in ways that ensure their continued imprint on the UCI as the central governance unit.

The impact of the UCI's male-dominated past reflects the “historical dynamic” of gendered field relations (Connell, 1987: 139). One former UCI director explained: “We know that positions [at the UCI] are often given … via political networks, [which] historically comes from a club in which it was men amongst themselves.” A former committee member stated in relation to men in international cycling governance, “I think there's too much history. I think there is still too many 50 to 60-year-old white men … old white dudes, basically. And they have history in [the] sport. They won races.” The durable legacy of historical inequalities reflects the significant advantages that, once gained, can continue to be retained by incumbents. At the same time, male incumbents are also in competition with one another. According to one former Director General, advancing through the ranks of cycling leadership “works via a craving for power, and here men are still currently warring amongst themselves.” That is, it is via not only coalition-building but also routine competition between incumbents that women are kept out of privileged positions.

### Key external actors and power relations

Beyond CFs and NFs, interviewees also pointed to sporting actors adjacent to the federated structure of cycling governance that regularly exert an influence on the actions of the UCI. The power relations between ASO and the UCI, for example, were illustrated during the COVID-19 pandemic, when the 2020 *Tour de France* was postponed. A UCI staff member explains: “The only ones who told the UCI, ‘Here are our dates, and that's it,’ was ASO!”, forcing the UCI to reschedule its own road world championships. In relation to gender equality actions, a former Director General stated, “One must put it simply: ASO has a lot of influence. And they said, we want to do women's cycling, but you’re [the UCI] the one who pays. We don’t want to lose money.” In this regard, the field-like nature of cycling governance at the meso-level, in which power is dispersed and no single actor wields absolute influence, allows key actors to minimize accountability on governance issues, including gender equality.

Yet interviewees also revealed that other adjacent actors can exert influence in ways that positively impact gender equality in cycling, most notably the IOC. A former Director General noted: “There aren't really any external forces or internal forces that would make them change [on gender]. One external force that could make them change is the IOC … [and] it does, actually, to be fair to the IOC. They've pushed very hard on gender equality.” While incumbents might attempt to resist this external influence and maintain the status quo, the financial and symbolic importance of the Olympic Games (and IOC influence over the Olympic programme) provides the IOC with leverage to push for certain gender equality goals. According to one coach and former WC member, “[the UCI] had a gun put to their head by the IOC saying, ‘you want to stay in the Olympics, you [must make cycling gender balanced]. Otherwise, you’re out.’” Off-field, the first woman appointed to the UCI Management Committee also occurred under pressure from the IOC, as one former Management Committee member explained: “in 1996 the IOC … asked all National Olympic Committees and all International Federations to have at least one woman on their [executive board]. Those who did not have one woman would be published on a blacklist.”

However, the contrast in aspiration between the playing field and leadership is striking: rather than gender parity, the IOC has only endorsed a minimum target of 30% for women in Olympic Movement leadership roles (IOC, 2021), also preferring the language of “target” to an enforceable quota (Adriaanse, 2017). Within the Olympic Movement, then, IOC actions reflect the interdependent relations between meso-level governing units, where most leaders have an interest in maintaining the male-dominated status quo. These adjacent field dynamics are then reflected in the gender regime of the UCI, further explaining how the position of incumbents has remained tightly coupled with men and masculinity over time. As we explore in the following section, this field environment is significant to understanding how international cycling has (and has not) shifted to accommodate specific gender equality initiatives and women leaders, which to date have not unsettled the male-dominated status quo.

## Part 2: Strategic management of the challenge of gender equality

### The women's committee as a managed field intervention

The 2010s saw the development of various initiatives and demands regarding female cycling.^3^ As part of its response to this mobilization, in 2013 the UCI established a WC, the role of which was to “advise the UCI and other Commissions on all matters related to women's cycling” (UCI, 2022). This reflects the committee structure as a preferred means by which IFs (and the IOC) channel the input of stakeholders and expand their capacity to address key governance issues. As one former Director General stated, “[IFs] work around commissions quite a lot, so you show your commitment [to gender equality] by creating a women's commission.” This move by the UCI mirrors a wider trend within the Olympic Movement, led by the IOC, which first established a Women and Sport Working Group in 1995. Questions have been raised over the effectiveness of so-called women's committees, given they can silo the work of gender equality (often removed from actual decision-making power) and can lack a critical understanding of gender (Matthews, 2021). In their study of World Athletics, Krech and colleagues (2022) ask whether such committees are a “token gesture,” given they are continually beset by roadblocks stemming from the sport's internal politics. We build on such work by situating the UCI WC within its field context, showing how meso-level relations matter fundamentally to the extent to which it could effect change.

The UCI established the WC at a time when the sport of cycling was facing what one former Director General referred to as “an existential crisis of governance” following various doping and corruption scandals (see also Plassard et al., 2021). Progress on gender equality was presented as demonstrating the “good governance” capabilities of the UCI at a time when existing field relations were at risk of collapse: “[it] really was about fundamental regime change. It was about breaking with the past … We believed passionately it needed [change].” Reflecting on their field-restoring role, however, the influence of commissions is limited, as noted by a former staff person: “The Commissions are always presented with the idea that they have power and that your advice can be powerful … [but] the Commission is essentially an advisory board. It has no decision-making power.” A former Director General similarly explained that the WC worked in an “apolitical way,” providing women in cycling with “a protected environment” where they could generate recommendations. Without a direct say in the actions of the UCI, a staff person explained that the WC could at best hope to “steer decisions.” They added, “in our case it helped create a strategic plan that guided the whole development of women's cycling.” The WC did therefore convince the UCI to put in place a strategic structure, the goal of which was to motivate the actions of field actors on gender equality, above all on the playing field.

Yet the agenda of the WC was rarely a priority for the UCI nor other actors within the field. As one interviewee noted, “[when the WC was established], that whole time was about the drugs and doping in the sport and there was a lot of nervousness around the actual promotion of cycling … So, the women's agenda seemed to take second fiddle even to that.” A former WC member referred to struggles amongst incumbents as a barrier to getting the attention of the UCI Management Committee:

You always had to be conscious of what you could sell. There was a political agenda [focused on] the reorganization of professional cycling and trying to wrestle power from the ASO … The concerns about women's racing were the two-minute discussion at the very end of the meeting.

As Connell has written, “a great deal of sexual politics is precisely about trying to make a latent interest salient in practice” (1987: 138). Anticipating the challenge of navigating existing field relations, WC leaders sought to create an effective coalition by bringing on board key professional cycling actors, including a representative of ASO. But according to one former staff member, the representative was “quite young and not necessarily the type of person we needed” to meaningfully influence ASO actions. One former WC member said the ASO put “someone on the committee just to protect their interests.” Thus, although a WC was in place, its work remained marginal to the core power struggles within the field––which centrally involved men and professional men's cycling.

In another attempt to influence the gendered character of field relations, the WC issued a survey of NFs aimed at documenting women's under-representation both on and off the playing field. However, a former WC member explained that “the uptake for that census was reasonably low,” demonstrating the “degree of apathy” amongst field actors towards the collection of data that might unsettle the status quo. This marginality extended to the women's cycling coordinator position, created in 2014. According to one staff person, “we were obliged to do something in that area, but it remains ‘fluff.’” Overall, the marginal impact of these formal actions on gender equality reflects the “state of play” within the meso-level SAF of international cycling: incumbents manage resistance from challengers in ways that block effective coalition-building and minimize disruptions to the field's gendered regime (Connell, 1987: 99). With the focus of gender equality interventions at the UCI defined as “women” rather than the persistent problem of male dominance and masculinity in cycling and its governance, incumbent men avoid scrutiny (Burton, 2015; Evans and Pfister, 2021).

### Women leaders as challengers

Alongside gender equality initiatives, numerous women have succeeded in securing leadership roles within the UCI, including Management Committee positions. Yet their experiences point to the costs of attempting to challenge the (gendered) status quo of the field as well as to the gendered basis of “social skill.” The first woman to serve on the Management Committee from 2000 to 2005 was Sylvia Schenck, who came to cycling via her role as a German politician.^4^ As an outside challenger, she adhered to different values compared with other field actors and had her reputation publicly attacked by the UCI when she revealed corrupt practices related to the 2005 UCI presidential election. Another interviewee and former committee member, who publicly critiqued the UCI for devaluing women's cycling, reported being told that such actions would not be tolerated. She added, “That's exactly how the UCI is … [T]he way they work with everyone within their cycling family, they’re all very connected.” She listed the professional (men's) cycling union and ASO as key allied “family members” who work together to protect the existing field order.

Another striking example is the case of Tracey Gaudry, the Chair of the WC and the first woman to serve as a UCI Vice President. For the WC, however, Gaudry's double role became a liability, since her increased status also made her a target within the sport's power struggles. According to one former staff person, “[O]nce the Commission became operational and Tracey Gaudry was very involved … then the power struggles began.” She added that Gaudry “was constantly being pushed out” of the inner circle of power brokers within the Management Committee. One former Director General observed that Gaudry “was an agitator,” who thought “I can make some change here” rather than accept the status quo. Ultimately, however, interviewees reported that Gaudry was removed from the drafting of the UCI's action plans for women in cycling, limiting her impact as a genuine challenger and undermining the voice and influence of the WC during the process.

Yet not all women at the UCI have been undermined, with our interviews suggesting that their acceptance is linked to whether and how they support the status quo. One female staff member reflected on the controversy surrounding Sylvia Schenck, saying: “I can tell you, I took note.” She explained her own contrasting approach to navigating the UCI's “political battles,” saying she has “always been to be loyal to the UCI.” The barriers to forming an effective alliance amongst women challengers were recognized by one former staff person: “you find two very different types of women [in sports leadership]: you find the women who make it and close the door behind them because they either enjoy being the only one sitting in that room full of dudes, or they are afraid that other women will be competition for them, and you have the women who bring other women with them.” Given the structural and symbolic disadvantage of challengers, individual women have less incentive to unite behind an alternative vision of the field, thereby leaving the male-dominated status quo intact.

Gender inequality on the playing field appears to play an important role in these field arrangements. As various former members noted, the strategic focus of the UCI WC was first and foremost on improving conditions for female riders in professional road cycling, leaving the under-representation of women in cycling leadership off the table. One former member observed: “Rightly or wrongly, we never considered [women in leadership roles] as part of our remit,” despite the efforts of the WC clearly being constrained by the sport's male-dominated leadership. He added: “You have to make the strategic decision about what's achievable, what's attainable and where you can have impact, given limited personnel, limited scope within the UCI, etc.” Spread thin by women's under-representation in professional cycling, and with women leaders blocked from disrupting existing field relations, the WC would not become a strategic intervention capable of advancing women's place in international cycling governance.

## Discussion and conclusion

This paper has argued that field context is a valuable meso-level analytical lens that can aid in explaining barriers to the advancement of gender equality in sports governance. Explaining the gender regime of the UCI, including what actions the organization is (and is not) willing to take on gender equality and the advancement of women leaders, requires attention to the interdependencies between the UCI and actors in surrounding and adjacent SAFs. The field dynamics of international cycling should be considered specific to the particular historical and contemporary dynamics of the sport. Moreover, even within the sport of cycling, field formation will be highly contextual and dependent on the issue at stake (Fligstein and McAdam, 2011). Nevertheless, we suggest that there is likely to be broad sociological utility to situating sports organizations within their meso-level and dynamic field context. The case of the UCI shows how the composition and organization of SAFs in sport can bear the imprint of gender, with a meso-level field approach aiding in explaining how relations of masculinity continue to be significant to how the sport of cycling is governed and by whom.

Our approach integrates and builds on the insights of two bodies of sports scholarship that have often been approached separately: that which explains the male dominance of sports organizations (e.g., Hoffman, 2011; Hovden, 2010; Piggott and Pike, 2019; Schull et al., 2013), and that concerned with the networked nature of sports governance (e.g., Chappelet, 2016; Geeraert et al., 2015; Meier and Garcia, 2021). By broadening our conceptualization of the meso-level––from the single organization to the field in which organizational actors interact––we have shown how sociologists of gender and sport can conceptualize the links between individual sports organizations and their gendered context, where context becomes not just the overarching macro-level gender order, but also relations between adjacent organizations (Burton, 2015; Evans and Pfister, 2021). Cycling offers an example of how the “boy's club” can be reproduced at the international level of sports governance (Hoffman, 2011; Schull et al., 2013), with the UCI serving as a central governance unit via which the historical accumulation of advantage to men is preserved. While our study was completed before the UCI disbanded its WC, this decision further suggests that the WC should be understood as an intervention aimed at managing women rather than meaningfully addressing gender inequality in cycling. When women leaders challenge existing field relations, the “cycling family” closes ranks, with incumbent actors acting strategically via the UCI to shore up the gendered status quo. As Fligstein and McAdam (2011) note, and as this study shows, challengers in this context may choose to “conform to the prevailing order” (p. 6), particularly where the individual costs of doing otherwise are high.

Centering strategic action as central to the dynamic nature of field relations in sport also expands existing accounts of sports governance. While the approach outlined in this paper builds on the notion of sports governance as a “horizontal, networked … web of relations” implicating “state, business and civil society actors” (Geeraert et al., 2015: 483), it adds an understanding of the boundaries and composition of these fields at the meso-level of sports governance, which is contingent and issue-specific, characterized by conflict, and underpinned by an unequal allocation of symbolic and material resources. Critically, our analysis of the UCI shows that to explain the coalitions and strategic actions that become possible in a given field of sports governance, it is necessary to consider how gender inequality and male dominance can be key to the power-laden relations between incumbents and challengers and to the role of governance units therein.

While the gender dynamics and strategic field relations that we have observed in the case of the UCI and the sport of cycling can by no means be considered representative of other fields of sports governance, this study does point to agendas for future research. Such research could consider how and why the field relations that shape outcomes for women and gender equality initiatives differ across sports, including those where challengers have had more success in reconfiguring relations of male dominance. Moreover, though not discussed in our analysis, the European bias of cycling is significant to understanding how the resources needed to successfully build coalitions are distributed within this and many other sports (Freeburn, 2012). Using a SAF approach to attend to the influence of other systems of difference-making and power, such as nation, region, and ability, can further enlarge how scholars engage with and explain mechanisms of stability and change in the meso-level dynamics of sports governance (Acker, 2006; Evans and Pfister, 2021).

## Notes

1. IFs also vary in terms of their age, budget, staffing capacity, and the popularity and commercial value of their sport (and disciplines).

2. The IOC redistributes one third of Olympic Games broadcasting rights to IFs, which are divided into groups A to E. The UCI, like many other federations, is financially heavily dependent on its retention in the B group.

3. For example, an international petition was launched in 2013 by women riders from the international peloton calling for women to have their own Tour de France (Masters, 2014).

4. Schenck is an Olympian from the sport of track-and-field.
